# Tricuspid valve papillary fibroelastoma in an adult patient: a case report and review of literature

**DOI:** 10.1186/s13019-025-03630-4

**Published:** 2025-11-28

**Authors:** Zhenchun Ji, Changhui Yu, Kadeerjiang Musha, Liangqiang Lin, Baihetiya Saimi, Abudusalamu Abudurexiti, Haoyue Huang, Zhenya Shen

**Affiliations:** 1 Department of Cardiovascular Surgery of the First Affiliated Hospital and Institute for Cardiovascular Science, Soochow University, No. 899 Pinghai Road, Suzhou, Jiangsu Province 215006 China; 2 Department of Cardiothoracic Surgery, People’s Hospital of Kizilsu Kirgiz Autonomous Prefecture, Kizilsu Kirgiz, Xinjiang, 845350 China

**Keywords:** Tricuspid valve papillary fibroelastoma, Diagnosis, Surgery, Case report

## Abstract

**Background:**

Tricuspid valve papillary fibroelastoma is a rare, benign primary cardiac tumor located on the tricuspid valve, with a potential risk of embolism. Here, we report a case of tricuspid valve papillary fibroelastoma successfully treated with surgical resection. The patient remained recurrence-free after a one-year follow-up.

**Case presentation:**

A 51-year-old female patient was admitted with a three-year history of chest tightness and shortness of breath. Transthoracic echocardiography revealed a 10 × 9 mm mass at the root of the right atrial surface of the anterior tricuspid valve leaflet, exhibiting movement in sync with valve opening and closing. The patient underwent surgical resection, and histopathological examination confirmed the diagnosis of papillary fibroelastoma.

**Conclusions:**

Tricuspid valve papillary fibroelastoma is still a rare benign cardiac tumor. Echocardiography serves as a reliable diagnostic tool. By reviewing the literature, we suggest that for tricuspid valve PFE with right-sided embolism and with a diameter greater than 5 mm, early surgical resection should be considered to prevent serious complications such as embolization.

## Introduction

Papillary fibroelastoma (PFE) is a slow-growing, benign cardiac tumor with an associated risk of embolism [1, 4]. Its etiology remains unknown, and it accounts for 8–16% of primary cardiac tumors [5, 7]. PFE predominantly affects cardiac valves, with the aortic valve being the most common site, while involvement of the tricuspid valve is rare [8]. Transthoracic and transesophageal echocardiography are the primary diagnostic modalities used to distinguish PFE from valvular vegetations. Surgical resection remains the standard treatment, aiming to preserve valve integrity and function. Given the risk of embolization, surgery is typically recommended unless contraindicated [9]. But there is no standardized treatment protocol for tricuspid valve PFE, the surgical indications is still controversial. Here, we present a case of tricuspid valve PFE and provide a comprehensive review of the literature to clarify the treatment options for this disease.

## Case presentation

A 51-year-old female patient was admitted with a three-year history of exertional chest tightness and dyspnea. She denied experiencing chest pain, palpitations, or fever. Her medical history included cranial cavernous angioma resection in 2019, and there was no family history of cardiac tumor. On physical examination, her temperature (T) was 36.7 °C, blood pressure (BP) was 118/79 mmHg, and heart rate was 71 beats per minute. Cardiac auscultation revealed no pathological murmurs.

**Fig. 1:**
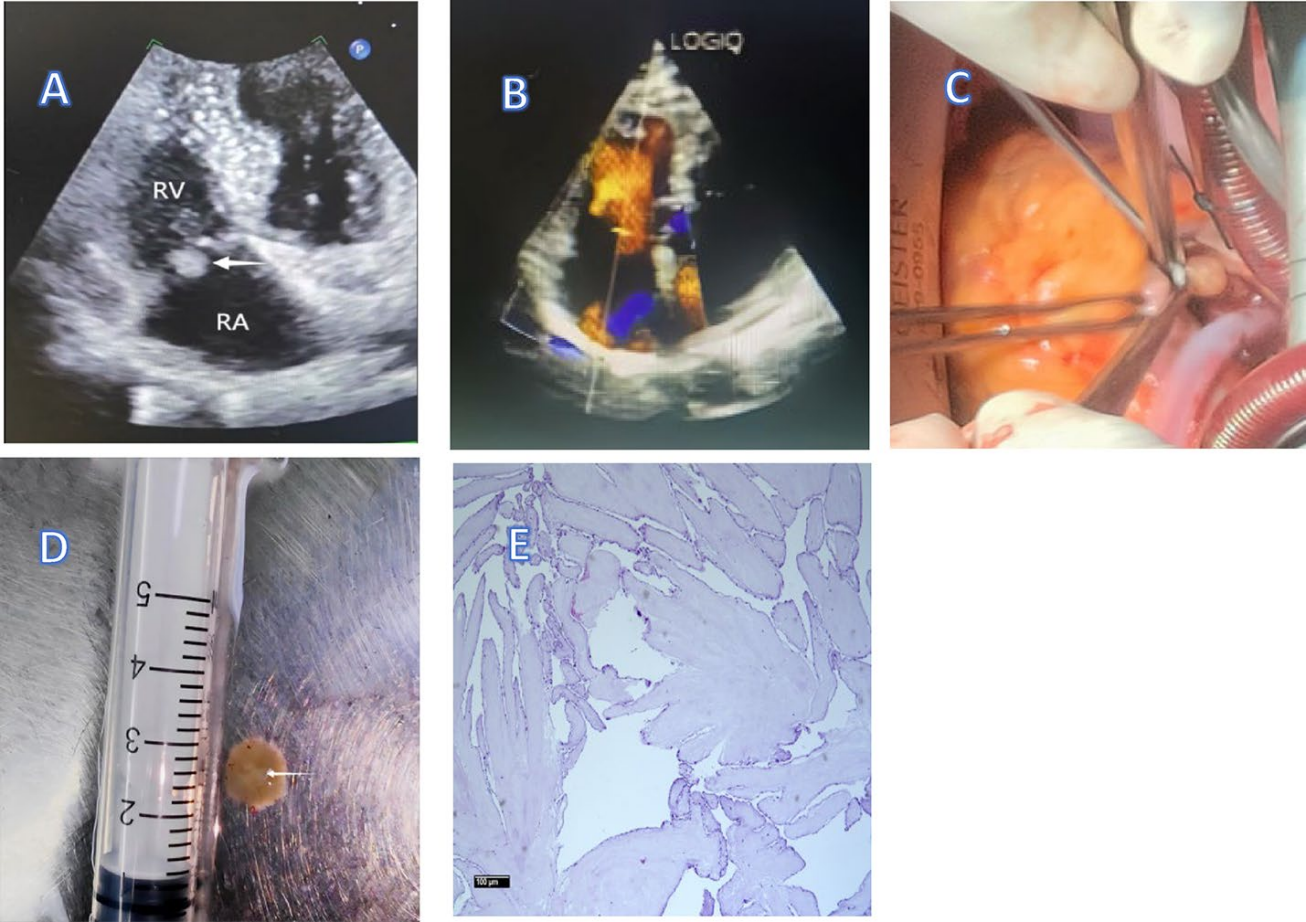
**(A)** Transthoracic echocardiography showed a mass at the root of the right atrial surface of the tricuspid valve anterior leaflet. The arrow indicates the mass,10mmX9mm large. **(B)** Ultrasound Color Doppler suggests mild tricuspid regurgitation. **(C)** Intraoperative photograph showing the tumor attached to the root of the right atrial surface of the anterior tricuspid valve leaflet. **(D)** Gross view of the tumor showing a gelatinous mass, 10mmX9mm large. The mass shows a villous structure in water, with an anemone-like appearance, the arrow indicates the brownish-white stalk. **(E)** Histopathological examination of the tumor showing an avascular central core of hyalinized connective tissue with papillary fronds, covered by a single layer of endocardial cells

Electrocardiography showed sinus rhythm without ST-segment changes. Transthoracic echocardiography (TTE) and transesophageal echocardiography (TEE) identified a 10 × 9 mm mass at the root of the right atrial surface of the anterior tricuspid valve leaflet (Figure 1A). The mass exhibited significant mobility without restricting leaflet opening or causing tricuspid valve mild insufficiency (Figure 1B). The pulmonary artery systolic pressure and mean pulmonary artery pressure estimated by echocardiography were 30 mmHg and 20 mmHg respectively. Coronary angiography revealed 40% stenosis in the proximal anterior descending artery. Pulmonary artery computed tomography angiography ruled out pulmonary embolism. Routine blood showed normal leukocyte levels, erythrocyte sedimentation rate and C-reactive protein levels.

The tricuspid valve mass was suspected to be a papillary fibroelastoma (PFE). Given its potential embolic risk, either from the mass itself or an associated thrombus, the patient underwent complete tumor excision easily on a beating heart under cardiopulmonary bypass, without cardioplegic arrest. The surgery was performed via median sternotomy. The ascending aorta, superior vena cava and inferior vena cava were cannulated to establish extracorporeal circulation. The cardio pulmonary bypass time was 35 min. Upon opening the right atrium, the tumor was found near the anterior tricuspid valve leaflet. It had a short stalk originating from the root of the anterior leaflet, which was easily fragmentable, with a mass diameter of approximately 10 mm and a pedicle measuring about 1 mm (Figures 1C and D). The tricuspid valve remained intact, with no evidence of closure insufficiency; therefore, tricuspid valvuloplasty was not performed. Postoperative histopathological examination confirmed the diagnosis of PFE (Figure 1E). The patient recovered well and was discharged on postoperative day 5. Follow-up echocardiography over the course of one year showed no tumor recurrence.

## Discussion

Primary cardiac tumors are rare, with PFEs being among the common types [40]. Approximately 6–15% of the PFEs occur on the tricuspid valve [19]. Most tricuspid valve PFEs are asymptomatic and are often incidentally detected during routine examinations. However, in some cases, they can cause severe symptoms of pulmonary embolism [26], presenting as sudden dyspnea and chest discomfort. Patients with tricuspid valve obstruction may experience lower limb edema and hepatic congestion. It is particularly important, as PFE embolism may be caused by small fragments of tumor or little thrombi and not necessarily by the entire tumor migration. There is an important difference between left- and right-sided localization of PFE. Systemic embolization (particularly to the central nervous system or coronary arteries) may be more dangerous compared to small pulmonary embolism, which may be asymptomatic, not causing significant complications. Additionally, PFEs can lead to arrhythmias, resulting in palpitations, dizziness, or syncope [41]. The case we reported primarily presented with nonspecific dyspnea, but no evidence of pulmonary embolism and tricuspid regurgitation was found. So the patient’s clinical symptoms may not be related to the tricuspid valve PFE and may be related to the coronary stenosis. The size of cardiac PFEs ranges from 2 to 70 mm, with most measuring around 10 mm in diameter [42], which aligns with the findings in our case.

Transthoracic echocardiography is routinely used for diagnosing tricuspid valve PFE. However, its sensitivity and specificity are low for tumors smaller than 2 mm. In such patients, transesophageal echocardiography should be performed, as it provides superior resolution, clearer visualization of the tumor-valve relationship, and greater utility in differential diagnosis [43]. Tricuspid valve PFE typically appears on echocardiography as a well-defined, small, mobile, pedicled mass attached to the tricuspid valve or tendon cords, with the potential to prolapse into the cardiac chambers during the cardiac cycle. Another characteristic feature is a “speckled or stippled” appearance due to peripheral echolucencies at the blood-tumor interface [41]. Cardiac computed tomography (CCT) and cardiac magnetic resonance (CMR) serve as secondary imaging modalities for diagnosing tricuspid valve PFE [25]. While CCT has limited resolution, CMR offers better tissue characterization and can aid in diagnosis [44]. Differentiation from thrombi and valvular vegetations is essential. Thrombi rarely form on valves, tend to be relatively fixed, and exhibit minimal mobility. In our case, the patient had no risk factors for infectious endocarditis, a normal leukocyte count, no fever, and negative blood cultures, making infective endocarditis unlikely. Differentiation from myxoma is also crucial, as myxomas are typically larger, cause significant obstruction, impair valve function, and have a distinct echocardiographic appearance [45]. Given these factors, we did not consider myxoma in this case.

Literature search in databases such as PubMed, Embase was conducted between 1983 and 2025, the search term was “papillary fibroelastoma” in combination with “tricuspid valve”. Including our case, a total of 69 reported cases of tricuspid valve PFE was identified. Of these, 33 cases were published in the last five years, accounting for nearly half of all reported cases. However, detailed data were unavailable for 37 cases (*n* = 10 [4,9], *n* = 6 [46], *n* = 5 [47], *n* = 2 [48], *n* = 1 [49], *n* = 2 [20], *n* = 1 [50]). The remaining 32 cases are summarized in Table 1. The ranged in age from 1 to 81 years old, with a median age of 63 years (interquartile range: 71.75–50.25). The male-to-female ratio was 17:15. Clinically, 10 cases were asymptomatic and detected incidentally, while 15 cases presented with dyspnea, seven with chest pain, three with fever, two with syncope, one with dizziness, and one with palpitations. These symptoms were nonspecific and not directly attributed to tricuspid valve PFE.

**Table 1:** Clinical Presentations of the Reported Cases and the Current Case

References	Age (years)	Sex	Clinical finding	Size(mm) or M(Q1-Q3)	Surgery	Comorbidity	Days in hospital	Follow-up(months)
Ali Ahmad et al. [10], 2024	43	M	None	6 × 5	TVP, MV repair, PVI, app ligation, Resection	AF	3	NR in 1
Kamel I et al. [11], 2024	60	M	Syncope	16 × 17	Resection	HTN; head injury	None	NA
Ku L et al. [12], 2024	31	M	None	14 × 12	Resection	None	7	NA
Chen D et al. [5], 2024	55	M	Chest pain	19 × 14	Resection	None	None	NA
Bashir H et al. [13], 2024	81	M	Chest pain; afebrile	13 × 12	CABG, AVR; Resection	CHD, HTN	None	NA
Wang Y et al. [14], 2023	73	M	None	8.4 × 6	TVP Resection	None	8	NR in 15
Actis Dato GM et al. [15], 2023	69	F	None	20 × 10	Resection	None	6	NA
Phan TQ et al., [16] 2023	17	F	Dyspnea	8.7 × 9.6	Resection	None	3	NR in 6
Zhang RS et al., [17] 2023	60	F	None	15 × 11	Resection by cardiac catheterization	DM; HTN; TR, ischemic stroke	NA	NA
Fang L et al. [18], 2022	51	M	Dyspnea	NA	TVR, Resection,	TR	NA	NA
Rana Y et al., [19] 2021	66	M	Dyspnea	17 × 13	Resection, PFO closure	HIV, DM, HTN, hyperlipidemia	3	NR
Kashiwagi Y et al., [20] 2021	62	F	Palpitation	23 × 17	Resection	RBBB, PE	NA	NA
Kavalerchyk V et al. [21], 2018	74	M	Chest pain, dyspnea	10 × 8	Conservative treatment	CHD	-	No change in 36
Artunduaga M et al. [22], 2017	1	F	None	15 × 15	TVP; Resection	None	6	NA
Rohani A et al. [23], 2017	64	f	Chest pain	9 × 6	Resection	TR	NA	NR in 6
Gollol-Raju NS et al. [24], 2016	71	M	Dyspnea	11 × 9.4	Resection by cardiac catheterization	Non-ischemic cardiomyopathy; HTN;	NA	NR in 1
Li W et al. [25], 2016	75	M	None	20 × 15	TVP, Resection	CHD, HTN, ischemic stroke	7	NR in 6
Choi KB t al [26], 2016	72	M	Dyspnea, afebrile	13 × 16	TVP; Resection	None	NA	NR
Shah RA et al. [27], 2015	65	M	Dyspnea	10 × 10 × 5	Resection	TR, PE, PH	NA	NR in 12
Srivatsa SV et al., [28] 2013	61	F	Dyspnea	13 × 9 × 11	Resection	TR, PH	NA	NR in 2
Karimi M et al. [29], 2013	8	M	Chest pain dyspnea	25 × 15	TVR; Resection	TR, PE	NA	NR
Karapanagiotidis GT et al. [30], 2012	81	F	Dyspnea, Dizzy spells	20 × 25	TVP; Resection	TR	7	NR in 3
Haron H et al. [31], 2012	68	M	Afebrile	10 × 10	MVR; TVP; Resection	DM; HTN; MI; TR	6	NR in 12
Massarenti L et al. [32], 2009	50	F	None	16 × 17	TVP; Resection	None	7	NA
Boodhwani M et al. [33], 2007	38	M	Dyspnea	15 × 20	TVP; Resection	NA	NA	NA
Mastroroberto P et al. [34], 2006	53	M	Chest pain; dyspnea	20 × 30	CABG; Resection	CHD, PE	NA	NA
Georghiou GP al. [35], 2003	67	F	Dyspnea	25 × 16	Resection	None	NA	NR in 4
Nishimura Y et al. [36], 1998	42	F	None	40 × 35	TVP; Resection	TR	NA	NR in 12
Neerukonda SK et al. [37], 1991	75	F	Dyspnea	1.5 × 2.0/ 33 × 2.0 × 18	TVP; Resection	PE	NA	NA
Wolfe JT 3rd et al. [38], 1991	75	F	None	27 × 27	CABG; Resection	Cerebral ischemic	15	Recurrence in 0.5
Frumin H et al. [39], 1983	64	F	Chest pain syncope	15 × 15	CABG; Resection	CHD	NA	NA
Current study	53	F	Dyspnea	10 × 9	Resection	None	5	NR in 12

M,male;F,female;TVP,tricuspid valvuloplasty;MV, mitral valve;PVI, pulmonary vein isolation;AF,atrial fibrillation;NR, non-tumor recurrence; NA,not available; HTN, hypertension;CABG, coronary artery bypass grafting; CHD,coronary artery disease；AVR, aortic valve replacement;DM, diabetes mellitus;TR,tricuspid regurgitation;TVR,tricuspid valve replacement;HIV,human immunodeficiency virus;PFO,patent foramen ovale;PE,pulmonary embolism;RBBB,right bundle-branch block;PH:pulmonary arterial hypertension;MI,mitral insufficiency;MVR, mitral valve replacement

Tumor size ranged from 5 × 6 mm to 40 × 35 mm, with no cases exceeding 40 mm. One case exhibited significant enlargement over a short period [37]. Tricuspid valve insufficiency was the most common comorbidity, and five cases were complicated by pulmonary embolism. Surgical resection, often combined with tricuspid valvuloplasty, was the primary treatment approach. Two cases were successfully treated *via* cardiac catheterization, a method that may warrant further consideration. One patient did not undergo surgery, and follow-up over three years showed no significant tumor progression [21]. The average hospital stay for surgical patients was 6.38 ± 3.09 days. There was one case of recurrence, but the patient died from a stroke 15 days postoperatively [21]. This raises the possibility that the tumor may not have been completely excised.

There is no standardized treatment protocol for tricuspid valve PFE, and clinical experience remains limited [20]. The surgical indications is still controversial. Some authors advocate for surgical resection as the primary treatment unless contraindicated [51], while others recommend resection in all cases due to the potential risk of embolic complications, regardless of tumor size [15]. Conversely, some suggest a conservative approach, reserving surgery for cases with significant obstructive symptoms or a high embolic risk [11]. In patients with elevated surgical risk, antiplatelet therapy may be considered, though supporting relevant evidence remains limited [43]. By reviewing the literature, most of the patients were treated surgically and only one patient was treated conservatively for coronary artery disease (CHD) reason. The minimum diameter of surgical resection was 5*6 mm, so we suggest that patients with right-sided embolism and tricuspid valve PFE with a diameter greater than 5 mm should be treated surgically. Otherwise, conservative treatment should be performed.

In our case, surgical resection was performed due to the tumor’s high mobility and risk of embolization. If the mass has a large pedicle or involves the valve, resection may require valve removal to prevent recurrence, followed by tricuspid valvuloplasty to restore normal blood flow. However, in our patient, the tumor had a small pedicle and was successfully excised without damaging the tricuspid valve leaflets, eliminating the need for valvuloplasty. Our team had considered a minimally invasive approach, such as aright mini-thoracotomy, However, considering the possibility of tricuspid valve repair and the risk of tumor detachment, we ultimately abandoned minimally invasive surgery.

As tricuspid valve PFE is a benign tumor, prognosis following resection is generally excellent, with postoperative recurrence being rare [42]. A case of mitral valve PFE recurrence was reported nine years after initial resection of a tricuspid valve PFE [52], but no recent recurrence cases have been documented. Our patient was followed for one year with no signs of recurrence, clinical symptoms disappeared.

## Conclusion

Tricuspid valve PFE is still a rare benign cardiac tumor. Echocardiography serves as a reliable diagnostic tool. By reviewing the literature, we suggest that for tricuspid valve PFE with right-sided embolism and with a diameter greater than 5 mm, early surgical resection should be considered to prevent serious complications such as embolization.

## Data Availability

No datasets were generated or analysed during the current study.

## References

[1] Ahmad A, El-Am EA, Kurmann RD, et al. Case report: a rare case of right-sided papillary fibroelastoma in a 1-year-old with congenital heart disease. Front Cardiovasc Med. 2020;7:624219. 10.3389/fcvm.2020.624219. 33585585 10.3389/fcvm.2020.624219 PMC7873290

[2] Ahmad A, Arghami A, El-Am EA, et al. Case report: a tale of a cardiac mass: looks like a papillary fibroelastoma, acts like a non-bacterial thromboendocarditis. Front Cardiovasc Med. 2021;8:782926. 10.3389/fcvm.2021.782926. 34869697 10.3389/fcvm.2021.782926 PMC8632806

[3] Kurmann RD, El-Am EA, Sorour AA, et al. Papillary fibroelastoma growth: a retrospective follow-up study of patients with pathology-proven papillary fibroelastoma. J Am Coll Cardiol. 2021;77(16):2154–5. 10.1016/j.jacc.2021.02.027. 33888255 10.1016/j.jacc.2021.02.027

[4] Ahmad A, El-Am EA, Kurmann RD, et al. Clinical and echocardiographic characteristics of patients with pathology proven right-sided papillary fibroelastomas. Int J Cardiol. 2022;349:123–6. 10.1016/j.ijcard.2021.11.083. 34871621 10.1016/j.ijcard.2021.11.083

[5] Chen D, Ma X, He Y, et al. Papillary fibroelastomas of tricuspid valve. Acta Cardiol. 2024. 10.1080/00015385.2024.2327139. 38469676 10.1080/00015385.2024.2327139

[6] Tyebally S, Chen D, Bhattacharyya S, et al. Cardiac tumors: Jacc cardiooncology state-of-the-art review. JACC Cardiooncol. 2020;2(2):293–311. 10.1016/j.jaccao.2020.05.009. 34396236 10.1016/j.jaccao.2020.05.009 PMC8352246

[7] Poterucha TJ, Kochav J, O’Connor DS, et al. Cardiac tumors: clinical presentation, diagnosis, and management. Curr Treat Options Oncol. 2019;20(8):66. 10.1007/s11864-019-0662-1. 31250250 10.1007/s11864-019-0662-1

[8] Maleszewski JJ, Bois MC, Bois JP, et al. Neoplasia and the heart: pathological review of effects with clinical and radiological correlation. J Am Coll Cardiol. 2018;72(2):202–27. 10.1016/j.jacc.2018.05.026. 29976295 10.1016/j.jacc.2018.05.026

[9] Mazur P, Kurmann R, Klarich KW, et al. Operative management of cardiac papillary fibroelastomas. J Thorac Cardiovasc Surg. 2024;167(3):1088–97. 10.1016/j.jtcvs.2022.06.022. 35989118 10.1016/j.jtcvs.2022.06.022

[10] Ahmad A, El-Am EA, Mazur P, et al. A case series of minimally invasive robotic-assisted resection of cardiac papillary fibroelastoma: the Mayo clinic experience. Mayo Clinic Proceedings: Innovations, Quality & Outcomes. 2024;8(2):143–50. 10.1016/j.mayocpiqo.2024.01.001. 10.1016/j.mayocpiqo.2024.01.001 PMC10905955 38434934

[11] Kamel I, Dietzius H, Magee T, et al. A case of rapidly growing tricuspid valve papillary fibroelastoma presenting with syncope. Cureus. 2024;16(3):e55510. 10.7759/cureus.55510. 38444927 10.7759/cureus.55510 PMC10912823

[12] Ku L, Chen Y, Ma X. A rare case of right-sided papillary fibroelastoma originating from the tricuspid valve. Acta Cardiol. 2024;1–2. 10.1080/00015385.2024.2313935. 10.1080/00015385.2024.2313935 38348873

[13] Bashir H, Ahmed A, Mahalwar G, et al. Tricuspid papillary fibroelastoma: a rare tumor in an uncommon location. Cureus. 2024;16(6):e61709. 10.7759/cureus.61709. 38975395 10.7759/cureus.61709 PMC11227620

[14] Wang Y, Saheb N, Husanova F, et al. Concomitant minimally invasive surgery for tricuspid valve papillary fibroelastoma and right lung cancer in an elderly patient: a case report and review of the literature. J Cardiothorac Surg. 2023;18(1):316. 10.1186/s13019-023-02431-x. 37950280 10.1186/s13019-023-02431-x PMC10638809

[15] Actis DG, Calia C, Lodo V, et al. A rare case of papillary fibroelastoma involving the tricuspid valve. A single center experience over a period of 22 years (1999–2021). Acta Chir Belg. 2023;123(5):563–5. 10.1080/00015458.2022.2064625. 35395925 10.1080/00015458.2022.2064625

[16] Phan TQ, Pham C, Bui V, et al. Minimally invasive resection of heart valve papillary fibroelastoma: two case reports and review of the literature. J Cardiothorac Surg. 2023;18(1):320. 10.1186/s13019-023-02392-1. 37957673 10.1186/s13019-023-02392-1 PMC10641953

[17] Zhang RS, Harari R, Kelly SM, et al. Percutaneous debulking of a tricuspid valve papillary fibroelastoma: a rare presentation and management approach. Circ Cardiovasc Imaging. 2023;16(12):e15970. 10.1161/CIRCIMAGING.123.015970. 10.1161/CIRCIMAGING.123.015970 38047386

[18] Fang L, Wu W, Wang J, et al. Flail tricuspid valve with torrential regurgitation caused by papillary fibroelastoma. Cardiol J. 2022;29(5):882–3. 10.5603/CJ.2022.0085. 36196660 10.5603/CJ.2022.0085 PMC9550330

[19] Rana Y, Tummala R, Engstrom K, et al. Discovery of tricuspid fibroelastoma on echocardiography. Cureus. 2021;13(8):e17359. 10.7759/cureus.17359. 34567899 10.7759/cureus.17359 PMC8453665

[20] Kashiwagi Y, Yoshida J, Itakura R, et al. Lung ventilation/perfusion scintigraphy shows the efficacy of anticoagulant therapy and surgical treatment for papillary fibroelastoma originating from the tricuspid valve. J Cardiol Cases. 2021;24(6):280–3. 10.1016/j.jccase.2021.04.022. 34917210 10.1016/j.jccase.2021.04.022 PMC8642632

[21] Kavalerchyk V, Staudt A, Stoebe S, et al. [Papillary fibroelastoma of the pulmonary and tricuspid valve in asymptomatic patients]. Dtsch Med Wochenschr. 2018;143(20):1484–8. 10.1055/a-0645-8559. 30286500 10.1055/a-0645-8559

[22] Artunduaga M, Jadhav SP, Eldin KW, et al. Radiopathologic correlation of a tricuspid valve papillary fibroelastoma detected in an infant. Radiol Case Rep. 2017;12(4):668–71. 10.1016/j.radcr.2017.08.001. 29484045 10.1016/j.radcr.2017.08.001 PMC5823306

[23] Rohani A, Bigdelu L, Nezafati M, et al. Three-dimensional echocardiography of a tricuspid valve papillary fibroelastoma. J Saudi Heart Assoc. 2017;29(1):57–9. 10.1016/j.jsha.2016.05.004. 28127220 10.1016/j.jsha.2016.05.004 PMC5247298

[24] Gollol-Raju NS, Joshi D, Daggubati R, et al. Successful biopsy and removal of a tricuspid valve papillary fibroelastoma in cardiac catheterization laboratory: a case report. Can J Cardiol. 2016;32(6):823–9. 10.1016/j.cjca.2015.06.032. 10.1016/j.cjca.2015.06.032 26577893

[25] Li W, Zheng J, Zhao H, et al. Beating-heart surgical treatment of tricuspid valve papillary fibroelastoma: a case report. Medicine. 2016;95(34):e4690. 10.1097/MD.0000000000004690. 27559977 10.1097/MD.0000000000004690 PMC5400344

[26] Choi KB, Kim HW, Kim DY, et al. Tricuspid papillary fibroelastoma mimicking tricuspid vegetation in a patient with severe neutropenia. Korean J Thorac Cardiovasc Surg. 2016;49(3):195–8. 10.5090/kjtcs.2016.49.3.195. 27298798 10.5090/kjtcs.2016.49.3.195 PMC4900863

[27] Shah RA, Kalidoss L, Mohanraj A, et al. Papillary fibroelastoma of tricuspid valve presenting as pulmonary embolism. Asian Cardiovasc Thorac Ann. 2015;23(7):858–60. 10.1177/0218492314526601. 24604555 10.1177/0218492314526601

[28] Srivatsa SV, Adhikari P, Chaudhry P, et al. Multimodality imaging of right-sided (tricuspid valve) papillary fibroelastoma: recognition of a surgically remediable disease. Case Rep Oncol. 2013;6(3):485–9. 10.1159/000355419. 24163665 10.1159/000355419 PMC3806706

[29] Karimi M, Vining M, Pellenberg R, et al. Papillary fibroelastoma of tricuspid valve in a pediatric patient. Ann Thorac Surg. 2013;96(3):1078–80. 10.1016/j.athoracsur.2012.12.043. 23992707 10.1016/j.athoracsur.2012.12.043

[30] Karapanagiotidis GT, Lees N, Howlett P, et al. Tricuspid valve papillary fibroelastoma: an unusual case of dizzy spells. Perfusion. 2012;27(2):156–9. 10.1177/0267659111431124. 22143091 10.1177/0267659111431124

[31] Haron H, Yusof MR, Maskon O, et al. Tricuspid valve papillary fibroelastoma: a rare tumor with a diagnostic dilemma. Heart Surg Forum. 2012;15(1):E59–60. 10.1532/HSF98.20111000. 22360910 10.1532/HSF98.20111000

[32] Massarenti L, Benassi F, Gallerano A, et al. Papillary fibroelastoma of the tricuspid anterior leaflet. J Cardiovasc Med (Hagerstown). 2009;10(12):933–5. 10.2459/JCM.0b013e32832fa0d1. 19623082 10.2459/JCM.0b013e32832fa0d1

[33] Boodhwani M, Veinot JP, Hendry PJ. Surgical approach to cardiac papillary fibroelastomas. Can J Cardiol. 2007;23(4):301–2. 10.1016/s0828-282x(07)70759-6. 17380224 10.1016/s0828-282x(07)70759-6 PMC2647888

[34] Mastroroberto P, Olivito S, Onorati F, et al. Papillary fibroblastoma of tricuspid valve with pulmonary embolization. Asian Cardiovasc Thorac Ann. 2006;14(3):e53. 10.1177/021849230601400328. 16714684 10.1177/021849230601400328

[35] Georghiou GP, Erez E, Vidne BA, et al. Tricuspid valve papillary fibroelastoma: an unusual cause of intermittent dyspnea. Eur J Cardiothorac Surg. 2003;23(3):429–31. 10.1016/s1010-7940(02)00761-3. 12614822 10.1016/s1010-7940(02)00761-3

[36] Nishimura Y, Naito Y, Fujiwara K, et al. Surgical treatment of a cardiac papillary fibroelastoma developing from the chordae of the tricuspid valve: report of a case. Surg Today. 1998;28(4):420–2. 10.1007/s005950050154. 9590710 10.1007/s005950050154

[37] Neerukonda SK, Jantz RD, Vijay NK, et al. Pulmonary embolization of papillary fibroelastoma. Arising from the tricuspid valve. Tex Heart Inst J. 1991;18(2):132–5. 15227497 PMC324981

[38] Wolfe JR, Finck SJ, Safford RE, et al. Tricuspid valve papillary fibroelastoma: echocardiographic characterization. Ann Thorac Surg. 1991;51(1):116–8. 10.1016/0003-4975(91)90464-2. 1985549 10.1016/0003-4975(91)90464-2

[39] Frumin H, O’Donnell L, Kerin NZ, et al. Two-dimensional echocardiographic detection and diagnostic features of tricuspid papillary fibroelastoma. J Am Coll Cardiol. 1983;2(5):1016–8. 10.1016/s0735-1097(83)80253-8. 6630754 10.1016/s0735-1097(83)80253-8

[40] Harling L, Athanasiou T, Ashrafian H, et al. Minimal access excision of aortic valve fibroelastoma: a case report and review of the literature. J Cardiothorac Surg. 2012;7:80. 10.1186/1749-8090-7-80. 22943845 10.1186/1749-8090-7-80 PMC3494536

[41] Nomoto N, Tani T, Konda T, et al. Primary and metastatic cardiac tumors: echocardiographic diagnosis, treatment and prognosis in a 15-years single center study. J Cardiothorac Surg. 2017;12(1):103. 10.1186/s13019-017-0672-7. 29183343 10.1186/s13019-017-0672-7 PMC5704631

[42] Gowda RM, Khan IA, Nair CK, et al. Cardiac papillary fibroelastoma: a comprehensive analysis of 725 cases. Am Heart J. 2003;146(3):404–10. 10.1016/S0002-8703(03)00249-7. 12947356 10.1016/S0002-8703(03)00249-7

[43] Tamin SS, Maleszewski JJ, Scott CG, et al. Prognostic and bioepidemiologic implications of papillary fibroelastomas. J Am Coll Cardiol. 2015;65(22):2420–9. 10.1016/j.jacc.2015.03.569. 26046736 10.1016/j.jacc.2015.03.569

[44] Bruce CJ. Cardiac tumours: diagnosis and management. Heart. 2011;97(2):151–60. 10.1136/hrt.2009.186320. 21163893 10.1136/hrt.2009.186320

[45] Hung LW, Lee CY, Hii HP, et al. Robot-assisted endoscopic removal of a huge tricuspid valve myxoma: case report. J Cardiothorac Surg. 2022;17(1):258. 10.1186/s13019-022-01978-5. 36203203 10.1186/s13019-022-01978-5 PMC9540697

[46] Mariscalco G, Bruno VD, Borsani P, et al. Papillary fibroelastoma: insight to a primary cardiac valve tumor. J Card Surg. 2010;25(2):198–205. 10.1111/j.1540-8191.2009.00993.x. 20149002 10.1111/j.1540-8191.2009.00993.x

[47] Ngaage DL, Mullany CJ, Daly RC, et al. Surgical treatment of cardiac papillary fibroelastoma: a single center experience with eighty-eight patients. Ann Thorac Surg. 2005;80(5):1712–8. 10.1016/j.athoracsur.2005.04.030. 16242444 10.1016/j.athoracsur.2005.04.030

[48] Anastacio MM, Moon MR, Damiano RJ, et al. Surgical experience with cardiac papillary fibroelastoma over a 15-year period. Ann Thorac Surg. 2012;94(2):537–41. 10.1016/j.athoracsur.2012.04.006. 22626753 10.1016/j.athoracsur.2012.04.006 PMC4329773

[49] Alozie A, Zimpfer A, Erbersdobler A, et al. Surgery for valvular and nonvalvular papillary fibroelastomas. Semin Thorac Cardiovasc Surg. 2022;34(2):560–8. 10.1053/j.semtcvs.2021.03.037. 34022368 10.1053/j.semtcvs.2021.03.037

[50] Kolek M, Dvorackova J, Motyka O, et al. Cardiac papillary fibroelastomas: a 10-year single-center surgical experience and long-term echocardiographic follow-up study. Biomed Pap Med Fac Univ Palacky Olomouc Czech Repub. 2020;164(1):84–91. 10.5507/bp.2019.053. 31748759 10.5507/bp.2019.053

[51] Zoltowska DM, Sadic E, Becoats K, et al. Cardiac papillary fibroelastoma. J Geriatr Cardiol. 2021;18(5):346–51. 10.11909/j.issn.1671-5411.2021.05.009. 34149823 10.11909/j.issn.1671-5411.2021.05.009 PMC8185441

[52] Hynes MS, Veinot JP, Chan KL. Occurrence of a second primary papillary fibroelastoma. Can J Cardiol. 2002;18(7):753–6. 12167963

